# The Effect of Orthodontic Therapy on Periodontal Health: A Review of the Literature

**DOI:** 10.1155/2014/585048

**Published:** 2014-05-29

**Authors:** Samah Alfuriji, Nora Alhazmi, Nasir Alhamlan, Ali Al-Ehaideb, Moatazbellah Alruwaithi, Nasser Alkatheeri, Amrita Geevarghese

**Affiliations:** ^1^College of Dentistry, King Saud bin Abdulaziz University for Health Sciences, Riyadh, Saudi Arabia; ^2^King Saud bin Abdulaziz University for Health Sciences, Dental Services-CR, King Abdulaziz Medical City-Riyadh, National Guard Health Affairs, P.O. Box 22490, Riyadh 11426, Saudi Arabia; ^3^College of Dentistry, King Saud bin Abdulaziz University for Health Sciences, KAMC, National Guard Health Affairs, Riyadh, Saudi Arabia; ^4^Saudi Speciality Certificate Program in Orthodontics, King Abdulaziz Medical City, National Guard Health Affairs, Riyadh, Saudi Arabia; ^5^Endodontic Division, King Abdulaziz Medical City, National Guard Health Affairs, Riyadh, Saudi Arabia; ^6^Department of Dental Public Health, College of Dentistry, King Saud bin Abdulaziz University for Health Sciences, KAMC, National Guard Health Affairs, Riyadh, Saudi Arabia

## Abstract

*Objectives.* This review aims to evaluate the effect of orthodontic therapy on periodontal health. *Data.* Original articles that reported on the effect of orthodontic therapy on periodontal health were included. The reference lists of potentially relevant review articles were also sought. *Sources.* A literature search was conducted using the databases, Medline, EMBASE, Cochrane Library, Web of Science, Google Scholar, and Scopus databases for relevant studies. The search was carried out by using a combined text and the MeSH search strategies: using the key words in different combinations: “periodontal disease,” “orthodontics” and “root resorption.” This was supplemented by hand-searching in peer-reviewed journals and cross-referenced with the articles accessed. Articles published only in English language were included. Letters to the Editor, historical reviews and unpublished articles were not sought. *Conclusions.* Within the limitations of the present literature review, it was observed that there is a very close inter-relationship between the periodontal health and the outcome of orthodontic therapy.

## 1. Introduction

Orthodontic treatment ensures proper alignment of the teeth and improves the occlusal and jaw relationship. This not only aids in better mastication, speech, and facial aesthetics, but also contributes to general and oral health, thereby improving the quality of life. Like any other treatment modalities, orthodontic treatment, in addition to its benefits, has also associated risks and complications. However, the risk and complication associated with treatment are reported to be considerably lower compared to other surgical or nonsurgical interventions [1].

However, the most commonly reported adverse effects of orthodontic treatment can be both local and systemic. This includes, tooth discolorations, decalcification, root resorption, periodontal complications, psychological disturbances, gastrointestinal complications, allergic reactions, infective endocarditis, and chronic fatigue syndrome [1–4]. It has been shown that orthodontic forces represent a physical agent capable of inducing an inflammatory reaction in the periodontium [5]. This reaction is necessary for orthodontic tooth movement [6]. One of the challenges of orthodontics is to finish the orthodontic treatment with the least effects on the root and periodontium.

This review aims to highlight the main coordinates of risk issues like periodontal complication and root resorption in orthodontics.

## 2. Periodontal Complication

Periodontal health is an important factor that may be used to evaluate the success of orthodontic therapy. Periodontal complications are reported to be one of the most common side effects linked to orthodontics [7]. Also, properly aligned teeth are easier to clean, and perhaps correct occlusion may promote healthier periodontium. The periodontal complications associated with orthodontic therapy mainly include gingivitis, periodontitis, gingival recession or hypertrophy, alveolar bone loss, dehiscences, fenestrations, interdental fold, and dark triangles [1, 2, 8, 9]. Presence of microbial plaque is reported to be the most important factor in the initiation, progression, and recurrence of periodontal disease in reduced periodontium [10].

The reasons behind these periodontal complications involve patient factors and the technique used in the treatment [11]. Patient factors include past periodontal condition, increased susceptibility, and poor oral hygiene. Smoking is also a known factor that affects the periodontal support [12, 13].

Orthodontic treatment and the procedures are known to induce both positive and negative local soft-tissue reactions in the gingiva. The negative reaction is mainly associated with gingivitis.

The presence of plaque is the considered as one of the main factors in the development of gingivitis [11, 12]. Orthodontic brackets and elastics might interfere with effective removal of dental plaque, thereby increasing the risk of gingivitis. Few clinical studies also reported poor periodontal health and greater loss of clinical attachment level distally in the dental arches. This could be a result of poor oral hygiene in molar regions and the presence of molar bands, which favors food lodgment [14]. However, as a result of the orthodontic treatment a shift in the composition and type of bacteria can be expected [15]. Orthodontic treatment is known to affect the equilibrium of oral microflora by increasing bacteria retention. In a study done by Ristic et al. [16] an increase in the value of periodontal indices and growth of periodontopathogenic bacteria were observed in adolescent patients undergoing fixed orthodontic treatment. In the majority of the patients, following placement of a fixed appliance, small amount gingival inflammation is visible, which could be transient in nature and does not lead to attachment loss [17]. Some reports support the fact that the fixed orthodontic treatment may result in localized gingivitis, which rarely progresses to periodontitis [18]. Gingival inflammation around orthodontic bands leads to pseudo-pockets, which usually disappear with debanding of the brackets. However, this is usually resolved within weeks of debanding. However, some of the published researches have reported reduced risk of gingivitis in the absence of plaque, orthodontic forces, and tooth movements [7, 19, 20]. If the orthodontic forces kept within the adequate limits in healthy reduced periodontal tissue support regions, the chances of gingival inflammation will be minimal [21]. Alexander [14] in his results has also reported lack of periodontal destruction over a longer period of time among patients wearing fixed appliances.

Published reports on human periodontal tissues state that the orthodontic banding performed with great care and proper maintenance of oral hygiene can prevent permanent periodontal destruction [22].

## 3. Pathophysiology

The periodontal ligament mainly consists of type I collagen, although type III collagen fibres are also present. The main function of PDL is sending proprioceptive signals to the brain and withstanding compressive forces during chewing movements. Various studies have reported significant recruitment of mononucleated cells, macrophages, dendritic cells, and MHC class II Ia-expressing cells in the pressure zone incident to orthodontic tooth movement [23, 24]. In the tension zone, however, minimal changes in the number and distribution of immune cells have been reported [25]. Under stress from the orthodontic treatment, there would be changes to the blood flow [26].

Neuropeptides are released from the periodontal nerve endings, which causes neurogenic inflammation in the compressed periodontal ligament [27]. Furthermore, various immunoregulatory molecules, such as interleukin-1 a, interleukin-6, and tumour necrosis factor-a, are released during inflammation and participate in the remodelling of the periodontium [28].

### 3.1. Root Resorption

One of the challenges of orthodontics is to finish the orthodontic treatment with the least effects on the root and periodontium. Root resorption is considered as undesirable but unavoidable iatrogenic consequence of orthodontic treatment [29]. Individual biologic variability, genetic predisposition, and the effect of mechanical factors are believed to influence apical root resorption [30, 31]. This undesirable complication of orthodontic treatment may result in tooth mobility and even permanent tooth loss [32]. It is an inflammatory process resulting in an ischemic necrosis in the periodontal ligament when the orthodontic force is applied [33]. It appears that apical root resorption is not just a result of orthodontic force but instead a combination of individual biological variability and the effect of mechanical factors [31].

Root resorption is a common consequence associated with orthodontic treatment. It has received considerable attention because of medicolegal exposure. It appears that apical root resorption results from a combination of individual biological variability and the effect of mechanical factors [31]. Loss of apical root structure is not predictable; when it progresses reaching the dentine is considered irreversible [34]. The cause of root resorption is still unknown, but the possible etiological factors are known and considered to be complex and multifactorial [30].

Severe root resorption after orthodontic treatment compromises the outcome of successful orthodontic treatment. Generally, root resorption can affect the longevity of the dentition. However, the majority of orthodontic root resorption does not affect the functional capacity of the dentition [35–37].

Studies of root resorption date back to more than 150 years. Bates in 1856 was the first to discuss root resorption of permanent teeth [38]. Several studies on root resorption have been published in the last 20 years [30, 31, 34, 39–41]. During this period, a better understanding of the process of root resorption has been achieved. The terms used to describe root resorption in the literature were variable like (root shortening, idiopathic root resorption, frequent complication, and common consequence).

Root resorption is defined as the destruction of the cementum or dentin by cementoclastic or osteoclastic activity; it may result in the shortening or blunting of the root [42]. Root resorption is also defined as microscopic areas of resorption lacunae visualized with histological techniques [43]. Root resorption is a general term that describes the general pathologic process which does not include any expression of the etiological factors.

Several etiological factors for root resorption are known (trauma, periodontal diseases, etc.), with almost similar outcome of root structure loss. Orthodontic root resorption is unique as compared to other types of root resorption. Brezniak and Wasserstein in 2002 suggested a new and more descriptive term of orthodontic root resorption based on the actual process and termed it orthodontically induced inflammatory root resorption (OIIRR) [44].

Orthodontically induced inflammatory root resorption (OIIRR) is a sterile inflammatory process that is extremely complex and composed of various disparate components including forces, tooth roots, bone, cells, surrounding matrix, and certain known biological messengers [44].

### 3.2. Prevalence and Diagnosis

Histological studies report greater than 90% occurrence of root resorption in orthodontically treated teeth with varying degree [45]; in most cases the loss of root structure is minimal and clinically insignificant. When diagnostic radiographic techniques are used lower percentages are reported of root resorption. Other studies reported that the average OIIRR is usually less than 2.5 mm when using panoramic or periapical radiographs [46, 47].

Using graded scales, OIIRR is usually classified as minor or moderate in most orthodontic patients. Severe resorption defined as exceeding 4 mm or 1/3 of the original root length, is seen in 1-5% of orthodontically treated teeth [48, 49]. The risk group where severe resorption may occur compromises one to three percent of the population [50].

Lupi et al reported the incidence of root resorption before orthodontic treatment 15% and after orthodontic treatment 73% [48].

### 3.3. Etiological Factors 

#### 3.3.1. Treatment Duration

Most studies have concluded that the risk and severity of external apical root resorption increase as the duration of orthodontic treatment increases [39, 44, 51–56]. Sameshima and Sinclair looked at a sample of 868 patients collected from 6 different specialist practitioners and found longer treatment times to be significantly associated with increased root resorption for maxillary central incisors [54]. The reasons for the longer duration in treatment may also have had an influence on the increased levels of root resorption seen in these patients.

However, others found no significant association between OIIRR and treatment duration [51, 57, 58].

Many variables are associated with treatment duration such as complicated treatment plans or lack of the patient compliance and these variables may also contribute to OIIRR.

#### 3.3.2. Appliance Type

Fixed appliances have been shown to cause more root resorption than removable appliances which can be explained by the increased range of tooth movement afforded by fixed appliances [52]. The risk of root resorption associated with different bracket designs has yielded inconclusive results [59, 60].

It is generally agreed that the use of a rapid maxillary expander is associated with increased levels of root resorption [61–64]. There are no other strong studies that investigated this correlation, but a case report has shown a significant OIIRR outcome with aligner treatment [44].

Kinzinger et al. studied the correlation between bonded Herbst functional appliance and root resorption. They concluded that banded Herbst appliance might deliver unphysiologic forces to immediate anchor teeth, thereby exposing these to a higher risk of root resorption than in other teeth incorporated into the anchorage either directly via bands or indirectly via occlusal or approximal contacts [65].

#### 3.3.3. Treatment Mechanics

When comparing straight wire and standard edgewise techniques, no statistically significant differences in the amount of tooth root loss or prevalence of root resorption were observed between groups [60]. Some studies have suggested that the Begg technique may induce more root resorption [60, 66, 67]. Other studies showed no significant difference between Begg, Tweed, or various straight wire edgewise techniques on root resorption [58, 59, 68].

L. Linge and B. O. Linge suggested that the use of intermaxillary elastics increased the amount of root resorption [52], but Sameshima and Sinclair did not find any correlation [54]. No difference has been found between the use of sectional and continuous mechanics [68].

Bioefficient therapy using contemporary orthodontic materials was found to produce less root resorption than the standard edgewise systems. The use of heat-activated and superelastic wires and a smaller rectangular stainless steel wire during incisor retraction and finishing played a role in this finding [69]. When comparing conventional edgewise systems to self-ligating systems, three studies concluded that there are no statistically significant differences in root resorption between systems [43, 70–72].

#### 3.3.4. Force Magnitude

Human and animal studies agree that there is an increase in severity of root resorption with increasing force magnitude [63, 73–78]. Harry and Sims used a scanning electron microscope to examine extracted human premolar teeth that had 50 g, 100 g, and 200 g of intrusive force. They concluded that higher forces increased root resorption through an increase in the stress to the root surface which increased the rate of lacunae development [74]. The more recent studies have confirmed that the higher forces increase the amount of external root resorption, thus confirming the previous studies. Chan and Darendeliler used a volumetric analysis of resorption craters on extracted human teeth to compare controls with a force of 25 g or 225 g, with buccal displacement [79] or intrusion [80]. Reitan, on the other hand, found that external root resorption was poorly correlated with force magnitude. He examined 72 premolars after application of 25 g to 240 g of intrusive, extrusive, and tipping movement over a period of 10 to 47 days [81].

A series of studies by Owman-Moll et al. agreed with the findings of Reitan. They looked at tooth movement with regard to force magnitudes of 50 g, 100 g, and 200 g. They found that there was a large interindividual variance, but no significant differences in the frequency and severity of root resorption could be detected. They concluded that root resorption was independent of force magnitude, but that individual reactions may be more important [82].

#### 3.3.5. Force Duration

Debate exists as to whether more root resorption is associated with continuous or intermittent forces. Many believe that discontinuous forces produce less root resorption because the pause in tooth movement allows the resorbed cementum to heal [83–88].

Acar et al. examined 22 human teeth. The patients were exposed to a continuous tipping force of 100 g on one side and on the other side an intermittent force was applied through elastics for 12 hours per day over a period of 9 weeks. Their results showed that the intermittent forces resulted in less root resorption. The accuracy of these results is questionable because the intermittent forces were subject to patient compliance [87].

Weiland [88] studied 84 premolars from patients which had been moved buccally with an orthodontic appliance. On one side of the mouth, force on the premolar was applied with a stainless steel wire (0.016 inch), while force on the contralateral premolar was applied with a superelastic wire (0.016 inch). Their results support the findings of Acar et al. [87] that continuous forces cause more resorption. They showed that the teeth activated with the super elastic wire moved significantly more but had 140% more resorption than the teeth with stainless steel wire.

Contrary to these reports Owman-Moll et al. [82] found no difference in the amount or severity of root resorption between forces applied continuously or intermittently after application of a buccally directed force of 50 g to human premolars.

#### 3.3.6. Direction of Tooth Movement

Intrusion has been consistently implicated as the most likely type of tooth movement to cause root resorption [57, 81]. Displacement of the root apex horizontally or torquing has been proven beyond doubt to produce root resorption [54, 89]. The highest incidence of root resorption is reported to occur when 3 to 4.5 mm of torquing movement was performed [54]. Reitan and Thilander and colleagues suggested that the stress distribution associated with tipping movements is more likely to cause root resorption than the stress distribution associated with bodily movement [64, 83].

#### 3.3.7. Amount of Tooth Movement

Sameshima and Sinclair [54] found that severe root resorption occurred in their samples when the root apex was displaced lingually, with a mean difference of 1 mm more than the control group. They concluded that root resorption is directly related to the distance moved by the tooth roots. Maxillary incisors tend to be moved more than other teeth in orthodontic treatment and therefore this is a possible explanation for why maxillary incisors are at a high risk of root resorption.

#### 3.3.8. Timing of Orthodontic Therapy

It is generally recommended that orthodontics be preceded by periodontal therapy based on the belief that orthodontics in the presence of inflammation can lead to rapid and irreversible breakdown of the periodontium [90]. Scaling, root planning (if necessary, by open flap debridement procedures for access), and gingival augmentation should be performed as appropriate before any tooth movement. The corrective phase of periodontal therapy, that is, osseous or pocket reduction/elimination surgery, ought to be delayed until the end of orthodontic therapy, because tooth movement may modify gingival and osseous morphology [91].

#### 3.3.9. Extraction

Sameshima and Sinclair [54] examined the relationship of the extraction pattern in detail as a factor affecting the resorption process. They concluded that extraction procedures (all first premolars, all second premolars, mandibular incisors, and asymmetric extractions) have the potential to produce root resorption during space closure. They observed a statistically significant difference in the resorption process when extraction and nonextraction groups were compared; among the extraction groups, the extraction of all first premolars showed the greatest resorption potential. Other studies that examined this factor did not find it to be significant [92, 93].

There are many etiological possible factors that may increase susceptibility for OIIRR. The current evidence available is conflicting and inconclusive. Weltman et al. [41] conducted a systematic review to this topic, where the factors have been grouped into likely, unlikely, and unclear risk-relationship categories.

## 4. Conclusion

Periodontal health is essential for any form of dental treatment. Adult patients must undergo regular oral hygiene instruction and periodontal maintenance in order to maintain healthy gingival tissue during active orthodontic treatment. Close monitoring of adults with reduced periodontal support is mandatory. Orthodontic treatment is usually contraindicated in patients with active periodontal disease or poor periodontal health as the chance of further periodontal deterioration is high in such case. Therefore, a thorough assessment of the periodontal health and level of attached gingival is recommended prior to the orthodontic treatment. Also, it is equally important to lay emphasis on the necessity of good oral hygiene in order to achieve the best treatment outcome. Oral hygiene instructions should be given before the start of orthodontic treatment and it should be reinforced during every visit.
